# Measuring loneliness at a national scale: The measuring loneliness in England (INTERACT) study’s approach and initial findings

**DOI:** 10.1186/s44263-026-00296-5

**Published:** 2026-08-06

**Authors:** Austen El-Osta, Aos Alaa, Mahmoud Al-Ammouri, Sami Altalib, Agustin Tristán-López, Azeem Majeed

**Affiliations:** Department of Primary Care and Public Health, School of Public Health, Imperial College London, London, W12 0BZ UK

**Keywords:** Loneliness, Social isolation, Social connection, Public health, Mental health, Social capital, Community health, COVID-19

## Abstract

**Background:**

Loneliness is increasingly recognised as a major public health challenge with significant implications for mental, physical and social wellbeing. While there were previous large scale studies including loneliness measures, few datasets capture loneliness across the full adult life course, integrate neighbourhood-level social capital and provide geospatial mapping at national scale. The Measuring Loneliness in England (INTERACT) Study was designed to address these gaps by characterising the distribution, intensity and sociodemographic patterning of loneliness within a large volunteer-based sample across diverse population groups in England. The aim of this paper is to describe the development, implementation and early findings of the INTERACT study.

**Methods:**

A total of 135,725 adults completed the online survey between March and July 2023. The instrument included validated measures of loneliness: the University of California Los Angeles (UCLA) 3 item loneliness scale and Office for National Statistics Direct Measure (DMOL), social capital indicators and demographic variables. Descriptive statistics were stratified by key subgroups. A geospatial analysis at Lower Super Output Area level was used to visualise clustering of loneliness across England.

**Results:**

135,725 participants across the full adult life course (16+) were included. The cohort was predominantly female (61.6%), White (82.6%) and aged over 65 (32.4%). The sample over-represented highly educated individuals and under-represented minority ethnic groups. On DMLO, 16.5% of participants reported feeling lonely “often or always,” while the UCLA-3 indicated that 21% to 22.5% frequently felt a lack of companionship, left out, or isolated (median score=6, Interquartile Range (IQR) = 3-7). Social capital perceptions were mixed (median=4, IQR) = 2-6). The COVID-19 pandemic exacerbated feelings of loneliness for 44.0% of respondents and isolation for 48.2%. As a volunteer-based, non-probability sample, findings are descriptive of respondents and are not intended to estimate population prevalence.

**Conclusions:**

The INTERACT Study provides a comprehensive national dataset on loneliness and social disconnection in England. The scale, methodological rigour and spatial granularity of the INTERACT study offer indicative insights that may inform the design of targeted, place-based interventions, pending confirmatory analyses in representative samples to mitigate loneliness and promote social connection across communities.

## Background

Loneliness is a subjective, distressing experience resulting from a discrepancy between desired and actual social relationships [1, 2]. It is distinct from social isolation, which refers to an objective lack of social contact [3]. Loneliness and social isolation are recognised as major global public health challenges, with substantial implications for mental, physical and social wellbeing. Extensive research demonstrated strong associations between loneliness and adverse health outcomes, including depression, anxiety, cognitive decline, cardiovascular disease, hypertension and increased mortality risk [1, 2, 4–6].

Recognising the growing burden, the World Health Organisation (WHO) identified social isolation and loneliness as critical determinants of health, highlighting the need for evidence-based interventions and policy responses [7, 8]. Beyond individual wellbeing, the economic burden of loneliness is equally profound, contributing to higher healthcare utilisation, lost workplace productivity and increased demand for social care services. In the United Kingdom (UK) [9], loneliness costs employers up to £3.7 billion annually or at least £9,976 per person per year [10, 11].

Loneliness is not confined to older adults as earlier studies identified a life-course distribution, often described as U-shaped, with peaks in adolescence and later life [12]. Young adults (16–25 years) consistently report high levels of loneliness [13], possibly due to academic stress, employment insecurity and increased digital socialisation, which may not always translate into meaningful social bonds [14, 15]. Meanwhile, in older populations, loneliness is frequently associated with bereavement, declining health and reduced social participation [16].

Geographic variation in loneliness is also well documented. Urban areas often report higher levels of loneliness despite greater population density, a phenomenon described as the “urban paradox” [17–19]. Pertinently, while some rural communities experience geographic isolation due to limited services and transport links, rurality does not inherently equate to social isolation. Evidence suggests that rural areas often benefit from stronger intergenerational networks and higher neighbourhood cohesion, which may buffer against loneliness compared with urban environments [20, 21]. Ethnicity and cultural background also play a significant role in loneliness. Some ethnic minority groups experience higher loneliness levels due to structural inequalities, language barriers, discrimination and weaker social integration [22]. Unsurprisingly, more recent studies have shown that the COVID-19 pandemic intensified loneliness and social isolation globally, with prolonged lockdowns, social distancing measures and economic disruptions contributing to worsening mental health outcomes [23–26].

Loneliness is a multidimensional construct influenced by individual, relational and structural factors, including personality traits, life transitions, socioeconomic conditions and community-level social capital [27]. Although loneliness is often conceptualised as comprising three major types (social, emotional and existential) its measurement as a phenomenon remains a significant methodological challenge. Validated loneliness scales such as the University of California Los Angeles (UCLA) 3-item Loneliness Scale [28] and the Office for National Statistics (ONS) Direct Measure of Loneliness (DMOL) [29] do not fully capture temporal dimensions of loneliness (e.g. duration, persistence, or fluctuation over time).

Neighbourhood-level factors play a crucial role in shaping social connectedness [30] but instruments like the UCLA Loneliness Scale and DMOL are not designed to assess constructs such as social capital, trust or cohesion [27], nor are they intended to do so. However, given that loneliness is influenced by both individual perceptions and community contexts, it is valuable to complement these scales with additional measures of social capital to support a more multidimensional understanding.

It is essential to distinguish related but non-equivalent constructs [31, 32]. Social capital, by contrast, refers to structural and cognitive features of social life such as trust, reciprocity and cohesion that enable access to resources and support [30, 33]. Similarly, social connection/disconnection functions as an umbrella construct describing the presence or absence of social ties and perceived belonging [8] while social support refers specifically to emotional, informational, or instrumental assistance from others. Therefore, although these constructs are related, they are not interchangeable since individuals may have extensive networks (low isolation) yet experience high loneliness if emotional closeness is lacking, whereas those with smaller networks but higher perceived trust may report lower loneliness, consistent with socioecological frameworks [34].

In the UK, loneliness was formally recognised as a national priority with the introduction of the Loneliness Strategy in 2018 [35]. Despite this, large-scale studies capturing loneliness across the full adult life course, alongside contextual factors such as social capital and geography, remain limited. Much of the existing literature has focused on older adults or small, localised cohorts, with less attention paid to younger populations or the broader social and structural determinants of loneliness [36, 37]. The Measuring Loneliness in England (INTERACT) Study was developed to address these critical gaps by deploying a large-scale national survey designed to characterise loneliness, social connection and related contextual factors across diverse population groups in England. By integrating validated loneliness measures with indicators of social capital, health status and socioeconomic context, INTERACT provides a multidimensional dataset for examining patterns of social disconnection in the post-pandemic period including the intensity (i.e. the severity and frequency) of subjective loneliness allowing for detailed subgroup analyses. Llinking individual-level responses to geographic units also enables exploration of spatial patterns in loneliness, including the identification of potential clustering across regions and such analyses may inform future place-based public health approaches.

The primary objective of the first phase of the INTERACT study was to characterise patterns of loneliness across population subgroups in England within a large, volunteer-based, non-probability sample. This initial paper in a programme of analyses provides a detailed account of the study design, survey instrument and sample characteristics, alongside initial descriptive findings from the March-July 2023 analytic cohort. These analyses are intended to describe the distribution and patterning of loneliness within the responding sample rather than to estimate population prevalence and to establish an empirical foundation for, longitudinal and policy-oriented investigations within the INTERACT programme.

## Methods

### Study design

The INTERACT study is a large-scale, cross-sectional, observational study designed to assess the distribution, sociodemographic patterning and geographic clustering of loneliness and social isolation within a large volunteer-based sample across England. The study employed a multi-methods quantitative approach, combining quantitative survey data with geospatial analysis to provide a comprehensive evaluation of loneliness and social connectedness within a large national volunteer cohort.

### Study setting and recruitment

The initial phase of the INTERACT survey was open from October 2021 to June 2024. The present manuscript reports descriptive findings from a defined subset of respondents who completed the survey between 1 March and 31 July 2023. Participants were recruited via National Healthcare Service (NHS) Primary Care Networks (PCNs) where invitations were sent via general practices and NHS Trusts using digital outreach methods, including emails and Short Message Service (SMS) messages. Voluntary and Community Sector Organisations facilitated recruitment through partnerships with local charities, community groups and national organisations focused on mental health and social wellbeing. The National Institute for Health and Care Research (NIHR) Be Part of Research (BPoR) Network advertised through research engagement platforms to enhance participation across diverse population subgroups. Targeted recruitment was conducted via Twitter (@LonelinessStudy, @ImperialSCARU), LinkedIn and institutional websites to reach younger adults and digitally engaged populations. Despite attempts to enhance diversity of participation, the final sample reflects a non-probability sample.

No quotas were imposed, but participation required access to an internet-enabled device (such as a smartphone, tablet or computer), which may have systematically excluded individuals without connectivity or digital literacy skills. Potentially eligible participants who received a link with an invitation to participate could learn more about the study by reading the participant information sheet (PIS) which could be accessed on the survey introduction page. Given that the resulting dataset constitutes a large, volunteer-based, non-probability sample, estimates derived from this dataset are not intended to represent population prevalence, but rather to describe patterns within the responding cohort.

### Inclusion and exclusion criteria

Participants were eligible to take part if they were 16 years or older, resided in England and were either registered as NHS patients or affiliated with NHS Trusts. The survey was open and participation was voluntary. Inclusion required participants to complete the online survey and to provide informed electronic consent before participation. Given the study’s focus on general population-based loneliness assessments, recruitment targeted a diverse range of individuals, including those from different age groups, ethnic backgrounds and socioeconomic statuses.

Exclusion criteria included individuals with severe cognitive impairments (e.g., dementia, schizophrenia or psychosis) that could compromise their ability to provide informed consent. Additionally, individuals receiving end-of-life care or experiencing severe mental health conditions requiring institutionalisation were excluded from the study. Participants who refused or were unable to provide informed consent were also ineligible. Given that the survey was conducted entirely online, individuals without internet access or digital literacy skills may have been inadvertently excluded, a limitation acknowledged in the study’s discussion.

### Survey development and measures

The INTERACT questionnaire was developed as a composite of various existing items, including validated loneliness measures, social capital indicators and community trust scales. The survey structure is shown in Table 1. Psychometric evaluation of the instrument, including Rasch analysis, is reported elsewhere [38].

**Table 1:** Survey Structure

Survey block	Description
Study introduction, link to Patient Information Sheet & Informed Consent form	Participants provided digital consent before proceeding.
Age, Gender & Postcode	Age, gender, full postcode
Loneliness Measures	UCLA Loneliness Scale, ONS Direct Measure of Loneliness (DMOL)
Social Connection	Frequency of contact with family and friends
Social Capital Indicators	Neighbourhood trust, community cohesion and perceived social support.
COVID	Covid specific questions
Demographics	Ethnicity, marital status, number of children, pet ownership, employment, disability and household size.
Consent to contact	Respondents could leave their contact details should they wish to volunteer to partake in a personal interview.

The final survey tool included 27 items over two pages, comprising 14 socio-demographic questions and 13 validated scale items to assess loneliness and social connectedness. Item selection was guided by conceptual relevance and prior use in validated instruments. To promote inclusivity, the survey was translated into 11 languages (the most used across England). Translations were conducted using forward/backward translation with review by native speakers. Respondents were able to review their responses before submission. The survey underwent pilot testing with 38 participants to assess clarity, usability and completion time leading to minor revisions for clarity and accessibility. The survey is included in Supplementary Material 1.

### Loneliness assessment

The online tool development and evaluation procedures were grounded in internationally recognised frameworks to ensure objectivity, validity and reliability, including the Consensus-based Standards for the Selection of Health Measurement Instruments (COSMIN) criteria for health-related instruments [39] and the psychometric guidelines outlined in the American Educational Research Associations, American Psychological Association and National Council on Measurement in Education (AERA-APA-NCME) Standards for Educational and Psychological Testing [40].

Loneliness was measured using two validated instruments that capture overall subjective loneliness but do not distinguish between social, emotional, or existential loneliness, nor do they assess temporal persistence. The UCLA Loneliness Scale (3-item version) [28] with the following three questions: *“how often do you feel that you lack companionship”*, *“How often do you feel left out”* and, “How often do you feel isolated from others”. Response options for this scale were: 1 (Hardly ever), 2 (Some of the time) and 3 (Often). Total scores range from 3 to 9, with higher scores indicating greater loneliness. The ONS Direct Measure of Loneliness (DMOL) [29] *“How often do you feel lonely”* question allowed response options: 1 (Never), 2 (Hardly ever), 3 (Occasionally), 4 (Some of the time) or 5 (Often or always) where higher scores indicate greater loneliness frequency.

The survey also incorporated social capital and community trust indicators adapted from validated scales: (i) the Neighbourhood Cohesion Scale [41, 42] and Social Trust Indicators [41]. These items assessed neighbourhood trust, reciprocity, cohesion, shared values and perceived social support. Participants rated agreement with statements such as “People in this neighbourhood can be trusted”, “*People around here are willing to help their neighbours*”, “*This is a close-knit neighbourhood where people generally know one another*”, and “*If I were sick, I could count on my neighbours to shop for groceries for me*”. Responses were dichotomised into “*agree or strongly agree*” (score=1) versus “*disagree or strongly disagree*” (score=0), with affirmative responses summed to generate an overall social capital score ranging from 0 to 7. Two negatively worded items were reverse coded.

### Data collection

The survey was hosted on the Qualtrics online platform, accessible via secure, encrypted links distributed through NHS, community networks and social media channels. No personally identifiable information (e.g., IP addresses) other than the full postcode was collected. Survey responses were pseudonymised at entry, stored on Imperial College London’s secure servers and handled in compliance with General Data Protection Regulation [43] and the Data Protection Act 2018 [44].

### Statistical analysis

Separate analyses were conducted for the UCLA Loneliness Scale, the DMOL and social capital score following ONS guidelines. Only completed surveys were analyzed. Analyses were descriptive. Frequencies and percentages were used for categorical variables and medians with interquartile ranges (IQR) for continuous variables. Participant characteristics were summarised using frequencies and percentages to provide a clear overview of the non-probability sample. Survey weights were not applied. Given the non-probability sampling design and absence of known selection probabilities, post-stratification weighting may introduce additional bias rather than correct it. Descriptive comparisons with national benchmarks are therefore presented for contextualisation only. Analyses were conducted separately for UCLA-3, DMOL and social capital indicators. Psychometric evaluation of the instrument, including Rasch modelling, is reported elsewhere [38]. All statistical analyses were performed using R software version 4.2.2.

### Geospatial analysis and heat mapping

To visualise the spatial distribution of loneliness across England within the geography of the UK, we employed kernel density estimation [45] using postcode-level data provided by participants. Responses were geocoded at the Lower Super Output Area (LSOA) level, allowing for aggregation while preserving anonymity. Kernel Density Estimation [45] was used to generate smoothed heat maps of the proportion of respondents reporting loneliness for both the UCLA Loneliness Scale and DMOL to visualise the spatial concentration of respondents reporting higher loneliness scores rather than to estimate population prevalence. Maps should therefore be interpreted as indicative of clustering within the sample. Areas with higher concentrations of participants reporting high loneliness scores were rendered as intensity “*hotspots*”. Geographic disparities were further examined in relation to rural-urban classifications and regional deprivation indices. Spatial analyses were conducted using QGIS (version 3.36.1 and R (version 4.2.2), an open-source geographic information system (GIS) software package for spatial data management, visualisation and mapping (QGIS Development Team, Open Source Geospatial Foundation Project, Beaverton, Oregon, USA), and R version 4.2.2.

### Contextualising the representativeness of the INTERACT sample

To contextualise the representativeness of the INTERACT sample, comparator estimates were derived from the 2021 Census for England using directly extracted data from relevant tables [46]. Descriptions and citations for each Census source are provided in the footnote of Table 2. Where England-only estimates were not readily available in published form for specific variables (e.g. sex and ethnicity), England and Wales estimates were used as the closest approximation. Comparator values were calculated using the closest available equivalent definitions, with categories aggregated or aligned as necessary to match study variables (e.g. age bands, legal partnership status, economic activity, and degree-level education). Comparisons were undertaken for key demographic characteristics including age, sex, ethnicity, marital status, educational attainment, employment status, and selected household variables where compatible measures were available. Variables without directly comparable Census definitions, or where alignment was not methodologically appropriate, were presented descriptively without comparator estimates. Given the non-probability sampling design, these comparisons are intended to provide descriptive context regarding sample composition rather than to support formal inference about population representativeness

**Table 2:** Respondent characteristics and national comparators

Variable (N = 135,725)	Study sample		Comparator		
Age	N	(%)	%	Difference from comparator (% points)	Source
16–25	8352	(6.2)	(14.6)	−8.41	TS007 England 2021
26–35	13,057	(9.6)	(16.8)	−7.18	TS007 England 2021
36–45	15,006	(11.1)	(15.8)	−4.76	TS007 England 2021
46–55	22,031	(16.2)	(16.5)	−0.3	TS007 England 2021
56–65	33,235	(24.5)	(14.9)	9.61	TS007 England 2021
>65	43,927	(32.4)	(21.4)	11.04	TS007 England 2021
Missing	117	(0.1)	(14.6)	−8.41	TS007 England 2021
**Sex**					
Female	83,661	(61.6)	(51.0)	-	TS008 E&W 2021
Male	50,729	(37.4)	(49.0)	-	TS008 E&W 2021
Would rather not say	498	(0.4)			
Other	804	(0.6)			
Missing	33	(0.0)			
**Ethnicity**					
Asian/Asian British	5162	(3.8)	(9.3)	-	TS021 E&W 2021
British Black/African/Caribbean	2310	(1.7)	(4.0)	-	TS021 E&W 2021
Mixed/Multiple ethnic groups	2025	(1.5)	(2.9)	-	TS021 E&W 2021
White	112,164	(82.6)	(81.7)	-	TS021 E&W 2021
White and Black Caribbean	462	(0.3)	(0.9)	-	TS021 E&W 2021
Other ethnic group	2722	(2.0)	(2.1)	-	TS021 E&W 2021
Missing	10,880	(8.0)			
**Marital status**					
Divorced	12,761	(9.4)	(9.1)	-	TS002 England 2021
In a relationship	12,404	(9.1)			
Married / Civil partnership	60,612	(44.7)			
Single	26,983	(19.9)	(37.9)	−13.4	TS002 England 2021
Widowed	9625	(7.1)	(7.1)	-	TS002 England 2021
Other	2992	(2.2)			
Missing	10,348	(7.6)			
**Highest level of education**					
A levels/College	36,700	(27.0)			
Secondary School	30,481	(22.5)			
University Degree or higher	58,008	(42.7)	(33.9)	12.41	TS067 England 2021
Missing	10,536	(7.8)			
**Employment status**					
Employed full-time	38,388	(28.3)			
Employed part-time	14,604	(10.8)			
Furloughed	54				
Retired	45,038	(33.2)			
Self-employed	7608	(5.6)			
Student (full or part-time)	3733	(2.8)			
Unemployed	7629	(5.6)	(3.5)	2.67	TS066 England 2021
Unpaid carer	1560	(1.2)			
Volunteer (full or part-time)	1459	(1.1)			
Other	4083	(3.0)			
Missing	11,569	(8.5)			
**Do you have any pets?**					
Yes	55,413	(40.8)			
No	69,895	(51.5)			
Missing	10,417	(7.7)			
**Number of people live in your household other than you**					
0	33,489	(24.7)			
1	52,760	(38.9)			
2–3	30,407	(22.4)			
4–5	7278	(5.4)			
More than 5	1494	(1.1)			
Missing	10,297	(7.6)			
**Do you have any children**					
Yes	84,561	(62.3)			
No	40,684	(30.0)			
Missing	10,480	(7.7)			
**Having children aged 16 years or under**					
Yes	19,287	(22.8)	(17.7)	-	TS012
No	64,991	(76.9)	(82.3)	-	TS012
Missing	283	(0.3)			
**Disability**					
Yes	24,915	(18.4)			
No	96,817	(71.3)			
Prefer not to say	3584	(2.6)			
Missing	10,409	(7.7)			
**Long-term conditions**					
Yes	62,038	(45.7)			
No	60,260	(44.4)			
Prefer not to say	3085	(2.3)			
Missing	10,342	(7.6)			

Comparator estimates were obtained from the 2021 Census for England or, where unavailable, England and Wales (E&W). Census categories were harmonised with study variables where necessary. Percentages are based on available data, with missing values reported separately. Variables without directly comparable Census measures are presented descriptively only. Comparisons are intended to contextualise sample composition and do not imply population representativeness. Census 2021 tables: TS007: Age by Single Year [62]; TS008: Sex [63]; TS021 Ethnic Group [64]; TS002: Legal Partnership Status [65]; TS067: Highest Level of Qualification [66]; TS066: Economic Activity Status [67]; TS012: Dependent Children in Household [68]

The study was reported in accordance with the Checklist for Reporting Results of Internet E-Surveys (CHERRIES) [47]; see Supplementary material 2.

## Results

### Participant characteristics

A total of 135,725 individuals participated in the INTERACT study. Respondent characteristics are presented in Table 2. Missing data across individual items ranged from approximately 7–9%.

The largest age group being those over 65 years old (32.4%), followed by those aged 56–65 (24.5%) and 46–55 (16.2%); Table 2. The majority of respondents were female (61.6%) and most identified as White (82.6%). In terms of marital status, 44.7% were married/ civil partnership, while 19.88% were single. Educational attainment was varied, with 42.7% holding a university degree or higher, 27.0% having A-levels/college education and 22.5% with secondary school as their highest level. Regarding employment, 33.18% were retired, 28.28% were employed full-time and 10.8% were employed part-time. 40.8% of respondents had pets and 62.3% had children, with 22.8% having children aged 16 or under. Additionally, 18.4% reported having a disability and 45.7% had a long-term health condition. Pet ownership was relatively common among participants, with 40.8% reporting that they had one or more pets (Table 2).

### UCLA loneliness scale and direct measure of loneliness (DMOL)

Participants reported varying levels of loneliness and social isolation as assessed by the UCLA Loneliness Scale and DMOL; Table 3. Regarding companionship (UCLA 1), 40.5% indicated feeling hardly ever or never lacking companionship, while 37.5% felt this way some of the time and 21.9% reported feeling often lonely. Similar patterns were observed for feeling left out (UCLA 2), with 37.6% hardly ever or never feeling left out, 41.4% feeling left out some of the time and 21% feeling left out often. For feelings of isolation (UCLA 3), 40.7% reported hardly ever or never feeling isolated, 36.8% felt isolated some of the time and 22.5% felt isolated often.

**Table 3:** Survey responses relating to loneliness, social connection and social capital

	N	(%)
**UCLA Loneliness Scale**		
**How often do you feel that you lack companionship (UCLA 1)?**		
Hardly ever or never	55,034	(40.5)
Some of the time	50,897	(37.5)
Often	29,791	(21.9)
*Missing*	3	(<0.01)
**How often do you feel left out (UCLA 2)?**		
Hardly ever or never	51,044	(37.6)
Some of the time	56,243	(41.4)
Often	28,435	(21)
*Missing*	3	(<0.01)
**How often do you feel isolated from others (UCLA 3)?**		
Hardly ever or never	55,180	(40.7)
Some of the time	50,009	(36.8)
Often	30,533	(22.5)
*Missing*	3	(<0.01)
**Direct Measure of Loneliness (DMOL)**		
Often or always	22,366	(16.5)
Some of the time	35,868	(26.4)
Occasionally	31,133	(22.9)
Hardly ever	30,122	(22.2)
Never	16,233	(12)
*Missing*	3	(<0.01)
**How many RELATIVES did you see or hear from in the last month?**		
0	7195	(5.3)
1	12,101	(8.9)
2	20,994	(15.5)
3 or 4	39,644	(29.2)
5–8	29,656	(21.9)
9 or more	15,825	(11.7)
*Missing*	10,310	(7.6)
**How many FRIENDS did you see or hear from in the last month?**		
0	11,936	(8.8)
1	13,850	(10.2)
2	19,188	(14.1)
3 or 4	31,679	(23.3)
5–8	25,299	(18.6)
9 or more	23,465	(17.3)
*Missing*	10,308	(7.6)
**To what extent do you agree or disagree with the following statements?**		
**Social Capital Scale**		
**People around here are willing to help their neighbours**		
Strongly disagree	9892	(7.3)
Disagree	28,505	(21)
Agree	70,185	(51.7)
Strongly agree	16,650	(12.3)
*Missing*	10,493	(7.7)
**This is a close-knit, or “tight” neighbourhood where people generally know one another**		
Strongly disagree	15,287	(11.3)
Disagree	48,439	(35.7)
Agree	51,770	(38.1)
Strongly agree	9037	(6.7)
*Missing*	11,192	(8.2)
**If I had to borrow 30 in an emergency, I could borrow it from a neighbour**		
Strongly disagree	37,211	(27.4)
Disagree	37,920	(27.9)
Agree	39,206	(28.9)
Strongly agree	9950	(7.3)
*Missing*	11,438	(8.4)
**People in this neighbourhood generally don’t get along with each other**		
Strongly disagree	24,389	(18)
Disagree	76,363	(56.3)
Agree	19,955	(14.7)
Strongly agree	3104	(2.3)
*Missing*	11,914	(8.8)
**People in this neighbourhood can be trusted**		
Strongly disagree	6222	(4.6)
Disagree	24,540	(18.1)
Agree	79,982	(58.9)
Strongly agree	12,849	(9.5)
*Missing*	12,132	(8.9)
**If I were sick, I could count on my neighbours to shop for groceries for me**		
Strongly disagree	15,711	(11.6)
Disagree	37,058	(27.3)
Agree	53,773	(39.6)
Strongly agree	17,547	(12.9)
*Missing*	11,636	(8.6)
**People in this neighbourhood do not share the same values**		
Strongly disagree	11,125	(8.2)
Disagree	65,576	(48.3)
Agree	40,057	(29.5)
Strongly agree	6482	(4.8)
*Missing*	12,485	(9.2)
**The COVID-19 pandemic & lockdowns made me feel more lonely**		
Strongly disagree	18,295	(13.5)
Disagree	45,953	(33.9)
Agree	38,662	(28.5)
Strongly agree	21,040	(15.5)
*Missing*	11,775	(8.7)
**The COVID-19 pandemic & lockdown made me feel more isolated**		
Strongly disagree	17,033	(12.5)
Disagree	40,937	(30.2)
Agree	43,513	(32.1)
Strongly agree	21,915	(16.1)
*Missing*	12,327	(9.1)

On the DMOL, 16.5% of participants reported feeling lonely often or always, 26.4% felt lonely some of the time, indicating a substantial burden of recurrent loneliness within the responding sample. Less than a quarter (22.9%) felt lonely occasionally and 22.2% rarely felt lonely. Only a small percentage (12%) reported never feeling lonely. These patterns should be interpreted in light of the study’s volunteer-based sampling design.

### Social connections among participants

Participants reported varying frequencies of contact with relatives and friends; Table 3.

### Neighbourhood dynamics, cohesion and trust

Participants’ perceptions of neighbourhood cohesion, trust and shared values are presented in Table 3 under the Social Capital Scale block. Participants reported mixed perceptions of neighbourhood cohesion and trust. While a majority agreed that neighbours were willing to help (51.7%) and could be trusted (58.9%), responses were more evenly distributed for indicators of close-knit communities, emergency support and shared values (Table 3).

### Impact of COVID-19 pandemic

During the COVID-19 pandemic, 44.0% of participants reported increased loneliness (agree/strongly agree) and 48.2% reported increased isolation (Table 3).

### Summary statistics for the UCLA loneliness scale and social capital score

Due to the non-normally distributed UCLA scores, we calculated the median and IQR. The median was 6 and IQR (3–7), with a minimum score of 3 and a maximum score of 9. Additionally, for the Social Capital Scale, the scores were not normally distributed. The median was 4 and IQR (2–6), with a minimum score of 0 and a maximum score of 7.

### Geospatial patterns of loneliness

**Fig. 1:**
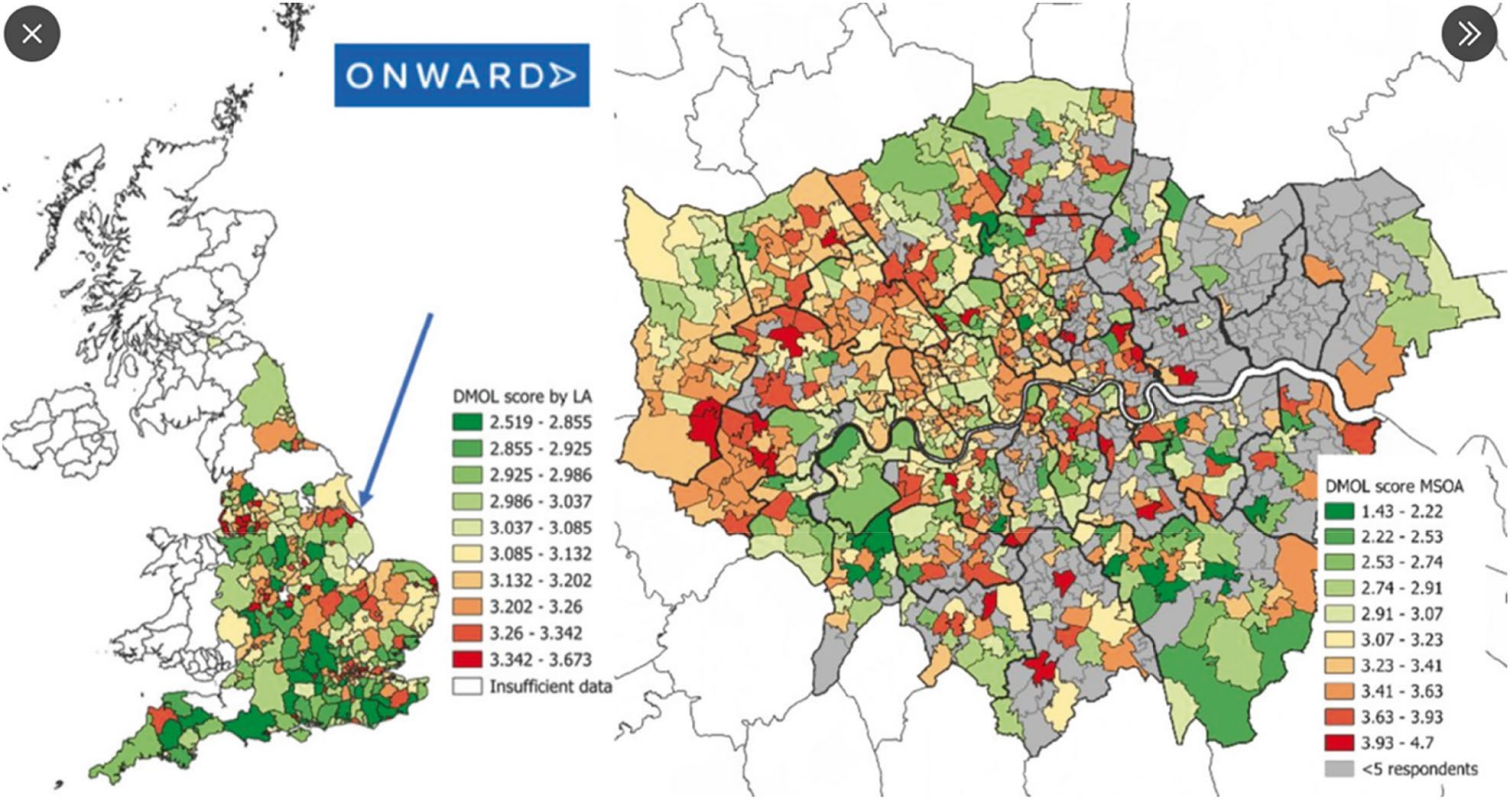
Geographic distribution of INTERACT study responses across England. The map displays England, with the United Kingdom shown for geographic context only; all data are restricted to respondents residing in England. Shaded areas represent the spatial distribution of participants reporting higher loneliness scores based on postcode-linked LSOA aggregation. Higher-density urban regions, including London (inset) and Birmingham, show greater concentrations of reported loneliness within the sample. These patterns reflect clustering of responses rather than population-level prevalence and should be interpreted as indicative of spatial distribution within a non-probability sample

Geospatial visualisation indicated clustering of participants reporting higher loneliness scores in major urban areas, including London, Birmingham and Manchester (Fig. 1). These spatial patterns reflect the distribution of responses within the sample rather than underlying population-level prevalence. Given the non-probability sampling design and variation in response density across regions, the observed clustering should be interpreted as indicative of within-sample concentration rather than geographic differences in loneliness across the wider population.

## Discussion

This study presents one of the largest descriptive investigations of loneliness and social disconnection in England based on a volunteer, non-probability sample. Drawing on data from over 135,000 community-dwelling adults, the primary value of the INTERACT dataset lies in its capacity to characterise patterns, subgroup variation and geographic clustering, rather than to estimate population parameters. Accordingly, the findings should be interpreted as descriptive of the responding cohort. While age was included as a covariate, this paper does not report age-stratified prevalence estimates.

A substantial proportion of participants reported experiencing loneliness, with 16.5% indicating feeling lonely “often or always,” and over two-thirds reporting loneliness at least occasionally. This pattern is further reflected in the distribution of UCLA-3 scores, where the median score was 6 (IQR 3–7). On this basis, at least half of respondents scored at or above 6, a threshold that has been used in some studies as an indicative marker of loneliness. However, such cut-offs are not universally standardised and should be interpreted with caution, particularly within non-probability samples. The observed distribution appears higher than estimates from population-based UK datasets, where loneliness is typically reported at approximately 10% using direct measures [48], although differences in measurement approach limit direct comparability. Importantly, the median represents the distribution of responses within the sample rather than a population prevalence estimate and should not be interpreted as indicating that 50% of the general population meets criteria for loneliness. Taken together, these findings indicate a right-shifted distribution of loneliness scores within the responding cohort rather than an elevated population burden.

This elevated distribution likely reflects a combination of sampling and measurement effects. Recruitment through NHS and community channels may have preferentially included individuals with poorer health or greater engagement with services, which may plausibly elevate observed levels of loneliness [24]. In addition, the UCLA-3, as an indirect multi-item scale, captures experiential aspects of social and emotional disconnection that may not be explicitly self-identified as loneliness and therefore tends to yield higher reported estimates than direct single-item measures [49].

Unsurprisingly, a substantial proportion of participants reported increased loneliness during the COVID-19 pandemic, consistent with previous findings [50]. However, these responses are based on retrospective self-report and may be influenced by recall and acquiescence bias. The cross-sectional design precludes attribution of changes in loneliness directly to pandemic-related factors, although the long-term implications of social disruption remain an important area for ongoing research [25].

Geospatial analysis identified clustering of higher loneliness scores in major urban centres such as London, Birmingham and Manchester. These findings are consistent with literature describing the ‘urban paradox’ whereby higher population density does not necessarily translate into greater social connectedness [22, 51]. However, given the non-probability nature of the sample, these patterns should be interpreted as reflecting response distribution rather than population-level geographic differences. While prior literature suggests urban–rural variation [19], the present study precludes inference regarding geographic patterns in the wider population.

Loneliness is increasingly recognised as affecting multiple age groups, including younger populations who may experience distinct socio-emotional pressures such as academic stress, employment insecurity and digital social comparison [15, 52]. Although the INTERACT study collected data form a large sample across England, our present analysis did not intend to provide age-stratified analyses and therefore does not establish differential risk across the life course. The findings instead support the conceptualisation of loneliness as a population-wide phenomenon, consistent with the WHO Commission on Social Connection [8]. By combining validated loneliness measures with a large sample, this study complements existing datasets such as the English Longitudinal Study of Ageing [53], which have predominantly focused on older populations [54]. Despite the current lack of population representativeness, INTERACT provides broader descriptive coverage across adult age groups. [53], which have predominantly focused on older populations [54]. Despite the current lack of population representativeness, INTERACT provides broader descriptive coverage across adult age groups.

The scale and geographic granularity of the INTERACT dataset provide a valuable resource for identifying population groups and communities that may benefit from targeted interventions. Although not population representative, the findings highlight the importance of considering both individual and neighbourhood-level determinants of loneliness when designing public health strategies to strengthen social connection and community resilience

The INTERACT Study represents one of the largest descriptive investigations of loneliness, social isolation and social capital conducted in England, with over 135,000 community-dwelling adults participating. This scale, although derived from a volunteer-based non-probability sample, enables detailed characterisation of patterns across demographic and health-related subgroups. The multi-channel recruitment strategy spanning NHS primary care networks, voluntary sector organisations, social media and national research platforms supported broad reach across population groups, although not population representativeness.

Methodologically, the study is anchored in the use of validated instruments, including the 3-item UCLA Loneliness Scale and DMOL, supporting comparability with existing literature. Since both measures are unidimensional assessments of subjective loneliness, the broader analytical perspective arises from their combination with conceptually distinct constructs such as social capital, neighbourhood trust and perceived cohesion. While these latter constructs are not dimensions of loneliness, their inclusion enables examination of contextual correlates of social disconnection. The use of geospatial kernel density estimation at LSOA level further extends the analysis by enabling visualisation of clustering within the sample.

A further strength is the study’s ability to provide a large-scale descriptive snapshot of loneliness and social connectedness. While the cross-sectional design precludes causal inference, it offers an empirical baseline for identifying patterns and generating hypotheses for subsequent investigation.

The principal limitation of this study is selection bias arising from the volunteer-based, non-probability sampling design. This is further reflected in the distribution of UCLA-3 scores, where the median suggests a right-shifted distribution of loneliness within the sample, reinforcing that observed levels should be interpreted as descriptive of the cohort rather than indicative of population estimates. Recruitment through NHS, voluntary sector and digital channels may preferentially include individuals who are more health-engaged or motivated to participate, potentially overrepresenting those with existing interest or experience of loneliness. Observed imbalances in age, gender and education further limit generalisability. In this context, the large sample size does not compensate for lack of representativeness.

We also acknowledge that additional bias may arise from structural exclusions especially that the online-only survey format may underrepresent individuals with limited digital access or literacy, including older adults, rural populations, people with disabilities and those experiencing severe social exclusion. Recruitment through institutional and digital networks may also miss individuals disengaged from both healthcare and community systems. Reliance on self-reported data also introduces potential for recall bias and social desirability bias, particularly given the stigma associated with loneliness. Retrospective items relating to the COVID-19 pandemic may also be influenced by recall and response biases, including acquiescence effects.

Pertinently, missing data (circa 7%) may introduce bias in descriptive estimates. While this was addressed in subsequent analyses using multiple imputation [55], complete case bias cannot be excluded in the present study.

Temporal factors may also influence findings, as data collection spanned multiple seasons during which loneliness may vary. Therefore, while we claim that the findings of the INTEACT study reflect real-world conditions, the temporal horizon may introduce variability related to seasonal or contextual influences.

It is notable also that although validated instruments were used, they do not capture important dimensions of loneliness, including chronicity, temporal fluctuation or distinctions between social, emotional and existential loneliness [56, 57]. As such, the findings reflect overall subjective loneliness rather than its specific forms or trajectories.

Finally, the absence of identifiable markers to preserve anonymity means that duplicate responses cannot be definitively excluded, although steps were taken to minimise this risk.

Despite these limitations, the INTERACT Study provides a large-scale descriptive dataset that complements existing probability-based surveys by offering greater scale, contextual breadth and spatial resolution. The findings should be interpreted as hypothesis-generating only.

Given the descriptive and cross-sectional nature of this study, the following implications are derived from observed patterns and should be interpreted as indicative and hypothesis-generating rather than causal or prescriptive. The INTERACT study provides large-scale descriptive evidence that loneliness is experienced across multiple age groups. While this aligns with existing literature, the present study does not provide age-stratified analyses and therefore does not establish differential risk across the life course. Nonetheless, the breadth of the sample supports consideration of approaches that address loneliness beyond older populations, including younger adults. Existing interventions, such as peer-based support and digital wellbeing approaches, may warrant further evaluation in this context, particularly given emerging evidence on the role of digital environments in shaping mental health and social connection [58, 59].

Observed differences in reported loneliness across ethnic groups highlight potential inequalities in social experience. However, as the study does not establish underlying mechanisms and is subject to sampling bias, these findings should be interpreted cautiously. They indicate a need for further research to better understand the drivers of these patterns and to inform the development of culturally appropriate and context-sensitive approaches, particularly in urban settings where diverse populations are concentrated.

The geospatial clustering of loneliness observed in major urban centres suggests that loneliness may exhibit spatial patterning within the sample. While this does not provide evidence of population-level geographic variation, it supports the potential value of spatial approaches for hypothesis generation and service planning. Place-based strategies, including those relating to urban design and public space, may be relevant areas for further investigation [60, 61].

The co-occurrence of lower social capital indicators and higher reported loneliness suggests that loneliness may be associated with broader social and contextual conditions. As social capital is conceptually distinct from loneliness, these findings should be interpreted as indicative of potential relationships rather than evidence of causation. They reinforce the importance of considering community-level factors, such as trust and cohesion, alongside individual-level approaches in future research and policy development. Interventions aimed at strengthening social cohesion may be promising but require empirical evaluation.

The reported impact of the COVID-19 pandemic on loneliness highlights the potential sensitivity of social connectedness to external shocks. However, these findings are based on retrospective self-report and should be interpreted with caution. They suggest that public health preparedness strategies may benefit from considering the potential for social disconnection during periods of disruption.

From a measurement perspective, the integration of loneliness and social capital indicators within a single large-scale survey demonstrates the feasibility of capturing both individual and contextual aspects of social disconnection. This may have implications for population surveillance. Existing monitoring frameworks, including the UK Government’s Loneliness Strategy and ONS wellbeing indicators, could be further developed to incorporate contextual measures, although the added value of such integration requires evaluation.

While the INTERACT platform was implemented in England, its structure and approach may be adaptable to other settings. However, further validation across different populations, health systems and cultural contexts would be required before broader application.

Taken together, these findings support the conceptualisation of loneliness as a population-level issue shaped by social and contextual factors. Addressing it is likely to require coordinated approaches across sectors, although the effectiveness of specific strategies requires further empirical investigation. The INTERACT Study provides a descriptive foundation for identifying patterns, populations and contexts that may warrant further targeted research and evaluation.

INTERACT is a cross-sectional study capturing post-pandemic reflections rather than a longitudinal COVID-19 cohort. The present analysis provides a descriptive baseline for subsequent work to further examine loneliness across populations and contexts. Building on these findings, future analyses will explore longitudinal patterns, potential determinants and intervention-relevant outcomes, as presented elsewhere [38].

Planned follow-up includes longitudinal data collection to assess how loneliness evolves over time in relation to life transitions, health status and social circumstances. Additional data collection among NHS staff will examine loneliness, burnout and team cohesion within a defined occupational cohort. Qualitative work is also planned to explore lived experiences of loneliness among selected groups, including young adults, ethnic minority populations, people with disabilities, carers and individuals experiencing social exclusion, with the aim of informing future hypothesis generation and intervention development.

Further work will examine the psychometric properties of the combined INTERACT instrument. Rasch modelling and qualitative analyses will be used to assess dimensionality, item performance and whether the current configuration adequately captures variation in subjective loneliness within a large and diverse sample. This work is not intended to redevelop established loneliness measures, but to evaluate how they function when integrated with additional contextual items. Findings have since been reported in detail [38].

These planned analyses aim to strengthen the measurement and analytical framework underpinning the INTERACT programme and to support its potential application in other populations, subject to appropriate validation.

## Conclusions

The INTERACT Study provides one of the largest descriptive assessments of loneliness and social capital in England to date. Given the non-probability sampling design, all findings are descriptive and hypothesis-generating and not intended to estimate population prevalence. Data from over 135,000 participants indicate substantial levels of reported loneliness and variation across demographic, geographic and contextual factors within the sample. These findings are consistent with the view that loneliness is shaped by a combination of individual and social conditions, although causal relationships cannot be inferred from the present study. The INTERACT dataset offers a large-scale empirical resource that complements existing probability-based surveys by providing greater scale, contextual breadth and spatial resolution. It provides a basis for further investigation of patterns, populations and contexts that may warrant targeted research and evaluation.

## Supplementary information

Supplementary material 1: INTERACT Survey Instrument Description

Supplementary material 2: CHERRIES Checklist Description

## Data Availability

Due to ethical and data protection constraints, individual-level data cannot be publicly shared. A de-identified dataset and accompanying codebook will be made available upon reasonable request from the corresponding author, subject to institutional approvals and data protection requirements.

## References

[1] Perlman D, Peplau L. Toward a social psychology of loneliness Personal relationships 3. Pers Relationsh Disord. 1981;3:31–43.

[2] Yanguas J, Pinazo-Henandis S, Tarazona-Santabalbina FJ. The complexity of loneliness. Acta Biomed. 2018;89:302–14. 29957768 10.23750/abm.v89i2.7404 PMC6179015

[3] Puyané M, Chabrera C, Camón E, Cabrera E. Uncovering the impact of loneliness in ageing populations: a comprehensive scoping review. BMC Geriatr. 2025;25:244. 40211165 10.1186/s12877-025-05846-4 PMC11984289

[4] Pyle E, Evans D. Loneliness-what characteristics and circumstances are associated with feeling lonely. Newport: Office for National Statistics; 2018.

[5] WHO. Social isolation and loneliness among older people. Geneva, Switzerland: World Health Organization; 2021.

[6] Department for Digital, C., Media & Sport, *Community Life Survey Technical Report 2019/20*. 2020.

[7] Goldman N, Khanna D, El Asmar ML, Qualter P, El-Osta A. Addressing loneliness and social isolation in 52 countries: a scoping review of National policies. BMC Public Health. 2024;24:1207. 38693471 10.1186/s12889-024-18370-8 PMC11061917

[8] Holt-Lunstad J. Social connection as a critical factor for mental and physical health: evidence, trends, challenges, and future implications. World Psychiatry. 2024;23:312–32. 39279411 10.1002/wps.21224 PMC11403199

[9] GOV.UK, *Population of England and Wales*. 2024.

[10] Michaelson J, Saamah Abdallah KJ *The cost of loneliness to UK employers*. 2017.

[11] Department for Digital, C., Media & Sport, *Loneliness monetisation report*. 2020.

[12] Hawkley LC, Buecker S, Kaiser T, Luhmann M. Loneliness from young adulthood to old age: explaining age differences in loneliness. Int J Behav Dev. 2022;46:39–49. 35001993 10.1177/0165025420971048 PMC8734589

[13] McGlone M, Long E. Are young adults with long-standing illness or disability at increased risk of loneliness? Evidence from the UK Longitudinal Household Study. J Public Health Res. 2020;9:1861. 33409244 10.4081/jphr.2020.1861 PMC7771026

[14] Orben A, Tomova L, Blakemore SJ. The effects of social deprivation on adolescent development and mental health. Lancet Child Adolesc Health. 2020;4:634–40. 32540024 10.1016/S2352-4642(20)30186-3 PMC7292584

[15] Twenge JM, Haidt J, Blake AB, McAllister C, Lemon H, Le Roy A. Worldwide increases in adolescent loneliness. J Adolesc. 2021;93:257–69. 34294429 10.1016/j.adolescence.2021.06.006

[16] Victor CR, Yang K. The prevalence of loneliness among adults: a case study of the United Kingdom. J Psychol. 2012;146:85–104. 22303614 10.1080/00223980.2011.613875

[17] Delhey J, Dragolov G. Happier together. Social cohesion and subjective well-being in Europe. Int J Psychol. 2016;51:163–76. 25683956 10.1002/ijop.12149

[18] Klinenberg E. Social isolation, loneliness, and living alone: identifying the risks for public health. Am J Public Health. 2016;106:786–7. 27049414 10.2105/AJPH.2016.303166 PMC4985072

[19] Matthews T, Danese A, Caspi A, Fisher HL, Goldman-Mellor S, Kepa A, et al. Lonely young adults in modern Britain: findings from an epidemiological cohort study. Psychol Med. 2019;49:268–77. 29684289 10.1017/S0033291718000788 PMC6076992

[20] Zhang X, Silverstein M. Intergenerational emotional cohesion and psychological well-being of older adults in rural China: A moderated mediation model of loneliness and friendship ties. J Gerontol B Psychol Sci Soc Sci. 2022;77:525–35. 34214164 10.1093/geronb/gbab122 PMC8893140

[21] Hussain B, Mirza M, Baines R, Burns L, Stevens S, Asthana S, et al. Loneliness and social networks of older adults in rural communities: a narrative synthesis systematic review. Front Public Health. 2023;11:1113864. 37255758 10.3389/fpubh.2023.1113864 PMC10225733

[22] Doyle DM, Barreto M. Stigma salience increases loneliness among ethnic minorities. Br J Soc Psychol. 2024;63:1625–39. 38558020 10.1111/bjso.12742

[23] El-Osta A, Alaa A, Webber I, Riboli Sasco E, Bagkeris E, Millar H, et al. How is the COVID-19 lockdown impacting the mental health of parents of school-age children in the UK? A cross-sectional online survey. BMJ Open. 2021;11:e043397. 33980516 10.1136/bmjopen-2020-043397 PMC8117442

[24] Bu F, Steptoe A, Fancourt D. Loneliness during a strict lockdown: Trajectories and predictors during the COVID-19 pandemic in 38,217 United Kingdom adults. Soc Sci Med. 2020;265:113521. 33257177 10.1016/j.socscimed.2020.113521 PMC7768183

[25] Groarke JM, Berry E, Graham-Wisener L, McKenna-Plumley PE, McGlinchey E, Armour C. Loneliness in the UK during the COVID-19 pandemic: Cross-sectional results from the COVID-19 Psychological Wellbeing Study. PLoS ONE. 2020;15:e0239698. 32970764 10.1371/journal.pone.0239698 PMC7513993

[26] Pierce M, Hope H, Ford T, Hatch S, Hotopf M, John A, et al. Mental health before and during the COVID-19 pandemic: a longitudinal probability sample survey of the UK population. Lancet Psychiatry. 2020;7:883–92. 32707037 10.1016/S2215-0366(20)30308-4 PMC7373389

[27] Lochner K, Kawachi I, Kennedy BP. Social capital: a guide to its measurement. Health Place. 1999;5:259–70. 10984580 10.1016/s1353-8292(99)00016-7

[28] Russell DW. UCLA loneliness scale (Version 3): reliability, validity, and factor structure. J Pers Assess. 1996;66:20–40. 8576833 10.1207/s15327752jpa6601_2

[29] Snape, D, Martin G, Measuring loneliness: guidance for use of the national indicators on surveys. Office for National Statistics, 2018.

[30] Nyqvist F, Victor CR, Forsman AK, Cattan M. The association between social capital and loneliness in different age groups: a population-based study in Western Finland. BMC Public Health. 2016;16:542. 27400659 10.1186/s12889-016-3248-x PMC4940959

[31] Wagner M, Rohwer WD. Age differences in the elaboration of inferences from text. J Educ Psychol. 1981;73:728.

[32] Holt-Lunstad J, Smith TB, Baker M, Harris T, Stephenson D. Loneliness and social isolation as risk factors for mortality:a meta-analytic review. Perspect Psychol Sci. 2015;10:227–37. 25910392 10.1177/1745691614568352

[33] Salisu I, Hashim N. A critical review of scales used in social capital research. IOSR J Bus Manag. 2017;19:34–40.

[34] McLeroy KR, Bibeau D, Steckler A, Glanz K. An ecological perspective on health promotion programs. Health Educ Q. 1988;15:351–77. 3068205 10.1177/109019818801500401

[35] GOV.UK, *A connected society: a strategy for tackling loneliness*. 2018.

[36] Inoue Y, Hamada H, Nakatani H, Ono I. Loneliness-associated factors among older adults: Focus on friendship type and number of friends. Jpn J Nurs Sci. 2025;22:e12649. 39828632 10.1111/jjns.12649 PMC11743425

[37] Luhmann M, Buecker S, Rüsberg M. Loneliness across time and space. Nat Rev Psychol. 2023;2:9–23. 36406179 10.1038/s44159-022-00124-1 PMC9640887

[38] Tristán-López A, Majeed A, El-Osta A. A Rasch-based psychometric evaluation of a 13-item loneliness and social connection scale in over 135,000 adults. Educ Methods Psychom. 2026;4:29.

[39] Mokkink LB, Prinsen CA, Bouter LM, Vet HC, Terwee CB. The COnsensus-based Standards for the selection of health Measurement INstruments (COSMIN) and how to select an outcome measurement instrument. Braz J Phys Ther. 2016;20:105–13. 26786084 10.1590/bjpt-rbf.2014.0143 PMC4900032

[40] Association, A.E.R., A.P. Association, and N.C.o.M.i. Education, *Standards for educational and psychological testing*. (No Title), 1985.

[41] Putnam, RD, Bowling Alone: America’s Declining Social Capital, in *Culture and Politics: A Reader*, L Crothers and C Lockhart, Editors. 2000, New York: Palgrave Macmillan US. p. 223-34.

[42] Hietasalo P, Seppä L, Lahti S, Niinimaa A, Kallio J, Aronen P, et al. Cost‐effectiveness of an experimental caries‐control regimen in a 3.4‐yr randomized clinical trial among 11–12‐yr‐old Finnish schoolchildren. Eur J Oral Sci. 2009;117:728–33. 20121937 10.1111/j.1600-0722.2009.00687.x

[43] GDPR, E., General data protection regulation (gdpr). Cit. on, 2018: p. 4.

[44] Legislation.gov.uk, *Data Protection Act 2018*. 2018.

[45] Murray CJL, et al. Global burden of 87 risk factors in 204 countries and territories, 1990–2019: a systematic analysis for the Global Burden of Disease Study 2019. Lancet. 2020;396:1223–49. 33069327 10.1016/S0140-6736(20)30752-2 PMC7566194

[46] Office for National Statistics, *Census-based statistics UK: 2021*. 2025.

[47] Eysenbach G. Improving the quality of Web surveys: the Checklist for Reporting Results of Internet E-Surveys (CHERRIES). J Med Internet Res. 2004;6:e34–e34. 15471760 10.2196/jmir.6.3.e34 PMC1550605

[48] Snape D, Manclossi S. Children’s and young people’s experiences of loneliness: 2018. UK: Office for National Statistics; 2018.

[49] Fisher RJ. Social desirability bias and the validity of indirect questioning. J Consum Res. 1993;20:303–15.

[50] Bu F, Steptoe A, Fancourt D. Who is lonely in lockdown? Cross-cohort analyses of predictors of loneliness before and during the COVID-19 pandemic. Public Health. 2020;186:31–34. 32768621 10.1016/j.puhe.2020.06.036 PMC7405905

[51] Friel S, Akerman M, Hancock T, Kumaresan J, Marmot M, Melin T, et al. Addressing the social and environmental determinants of urban health equity: evidence for action and a research agenda. J Urban Health. 2011;88:860–74. 21877255 10.1007/s11524-011-9606-1 PMC3191214

[52] Dibb B, Foster M. Loneliness and Facebook use: the role of social comparison and rumination. Heliyon. 2021;7:e05999. 33521361 10.1016/j.heliyon.2021.e05999 PMC7820562

[53] Brauer M, et al. Global burden and strength of evidence for 88 risk factors in 204 countries and 811 subnational locations, 1990–2021: a systematic analysis for the Global Burden of Disease Study 2021. Lancet. 2024;403:2162–203. 38762324 10.1016/S0140-6736(24)00933-4 PMC11120204

[54] Steptoe A, Breeze E, Banks J, Nazroo J. Cohort profile: the English longitudinal study of ageing. Int J Epidemiol. 2013;42:1640–8. 23143611 10.1093/ije/dys168 PMC3900867

[55] El-Osta A, Al-Ammouri M, Alaa A, Altalib S, Tristán-López A, Majeed A, *Unpacking the Predictors of Loneliness: An Inferential Analysis from the INTERACT Study*. 2025, Research Square.

[56] De Jong-Gierveld J, van Tilburg TG. A 6-item scale for overall, emotional, and social loneliness: Confirmatory tests on survey data. Res Aging. 2006;28:582–98.

[57] Yang M, Wei W, Ren L, Pu Z, Zhang Y, Li Y, et al. How loneliness linked to anxiety and depression: a network analysis based on Chinese university students. BMC Public Health. 2023;23:2499. 38093295 10.1186/s12889-023-17435-4 PMC10720215

[58] Keles B, Niall M, Grealish A. A systematic review: the influence of social media on depression, anxiety and psychological distress in adolescents. Int J Adolesc Youth. 2020;25:79–93.

[59] Magid K, Sagui-Henson SJ, Sweet CC, Smith BJ, Chamberlain CE, Levens SM. The impact of digital mental health services on loneliness and mental health: results from a prospective, observational study. Int J Behav Med. 2024;31:468–78. 37488324 10.1007/s12529-023-10204-y PMC11106110

[60] Qi J, Mazumdar S, Vasconcelos AC. Understanding the relationship between urban public space and social cohesion: a systematic review. Int J Community Well-Being. 2024;7:155–212.

[61] Zhang Y, Chen G, He Y, Jiang X, Xue C. Social interaction in public spaces and well-being among elderly women: towards age-friendly urban environments. Int J Environ Res Public Health. 2022;19:746. 35055567 10.3390/ijerph19020746 PMC8775950

[62] Office for National Statistics, *Age by single year*. 2023.

[63] Office for National Statistics, *Sex*. 2023.

[64] Office for National Statistics, *Ethnic group*. 2023.

[65] Office for National Statistics, *Legal partnership status*. 2023.

[66] Office for National Statistics, *Highest level of qualification*. 2023.

[67] Office for National Statistics, *Economic activity status*. 2023.

[68] Office for National Statistics, *Country of birth (detailed)*. 2023.

