# Supplementary material for: Measuring loneliness at a national scale: The measuring loneliness in England (INTERACT) study’s approach and initial findings
Source: BMC Glob Public Health. 2026 Aug 6;4:69. doi: 10.1186/s44263-026-00296-5 (PMC13445768; doi:10.1186/s44263-026-00296-5)
Supplement: Supplementary file 1 [file 44263_2026_296_MOESM1_ESM.docx]

INTERACT ICREC (NWL) Generic community facing

Start of Block: Default Question Block

Q1 Imperial College London is conducting a research study to help identify ways to tackle social isolation & loneliness in the community setting. Please consider participating in this brief online questionnaire which should not take more than 10 minutes to complete. You can choose the preferred language for completing this questionnaire using the drop-down list on the top-right of page. All your responses will be  anonymous.   For more information, please refer to the Participant Information Sheet.   For information on Social Isolation & Loneliness helplines, please refer to the following link. Code: Community Generic

Q2 Please confirm that you consent to participate in this survey.

- Yes (1)
- No (2)

Q3 What is your full postcode? (We will not be using this information to identify you)

________________________________________________________________

Q4 How often do you feel that you lack companionship?

- Hardly ever or never (1)
- Some of the time (2)
- Often (3)

Q5 How often do you feel left out?

- Hardly ever or never (1)
- Some of the time (2)
- Often (3)

Q6 How often do you feel isolated from others?

- Hardly ever or never (1)
- Some of the time (2)
- Often (3)

Q7 How often do you feel lonely?

- Often or always (5)
- Some of the time (4)
- Occasionally (3)
- Hardly ever (2)
- Never (1)

Q8
What is your age? (in years)

________________________________________________________________

Q9 What is your gender?

- Male (1)
- Female (2)
- Other (11)
- Would rather not say (10)

| Page Break |  |
| --- | --- |

Q10
How many RELATIVES did you see or hear from in the last month?

- 0 (none) (1)
- 1 (2)
- 2 (3)
- 3 or 4 (4)
- 5-8 (5)
- 9 or more (6)

Q11
How many FRIENDS did you see or hear from in the last month?

- 0 (none) (1)
- 1 (2)
- 2 (3)
- 3 or 4 (4)
- 5-8 (5)
- 9 or more (6)

Q12 To what extent do you agree or disagree with the following statements?

|  | Strongly disagree (1) | Disagree (2) | Agree (3) | Strongly agree (4) |
| --- | --- | --- | --- | --- |
| People around here are willing to help their neighbours (Q12_matrix_1) |  |  |  |  |
| This is a close-knit, or ‘‘tight’’ neighbourhood where people generally know one another. (Q12_matrix_2) |  |  |  |  |
| If I had to borrow £30 in an emergency, I could borrow it from a neighbour. (Q12_matrix_3) |  |  |  |  |
| People in this neighbourhood generally don’t get along with each other. (Q12_matrix_4) |  |  |  |  |
| People in this neighbourhood can be trusted. (Q12_matrix_5) |  |  |  |  |
| If I were sick I could count on my neighbours to shop for groceries for me. (Q12_matrix_6) |  |  |  |  |
| People in this neighbourhood do not share the same values (Q12_matrix_7) |  |  |  |  |
| The COVID-19 pandemic & lockdowns made me feel more LONELY (Q12_matrix_8) |  |  |  |  |
| The COVID-19 pandemic & lockdown made me feel more ISOLATED (Q12_matrix_9) |  |  |  |  |

Q13 What is your employment status?

▼ Employed full-time (1) ... Other (please specify) (9)

Q14 What best describes your ethnic group or background?

- White (1)
- Mixed/Multiple ethnic groups (34)
- White and Black Caribbean (35)
- Asian/Asian British (36)
- British Black/African/Caribbean (37)
- Other ethnic group (please specify) (38) __________________________________________________

Q15 What is your marital status?

- Married / Civil partnership (1)
- Single (2)
- In a domestic relationship (3)
- Divorced (5)
- Widowed (6)
- Other (please specify) (7) __________________________________________________

Q16 Do you have any pets?

- Yes (2)
- No (3)

Q17 What is your highest level of education?

- Secondary School (1)
- A levels/College (2)
- University Degree or higher (3)

Q19 Do you have any children?

- Yes (1)
- No (2)

Display this question:

If Do you have any children? = Yes

Q20 Are any of your children aged 16 years or under?

- Yes (1)
- No (2)

Q18 How many people other than you live in your household?

- 0 (2)
- 1 (6)
- 2-3 (3)
- 4-5 (4)
- More than 5 (7)

Q21 Do you have a disability?

- Yes (5)
- No (6)
- Prefer not to say (3)

Q22 Do you have any long-term health conditions (e.g. mental health, diabetes, asthma)?

- Yes (1)
- No (4)
- Prefer not to say (6)

Q23 Thank you for taking the time to answer our survey.  We are looking to interview up to 30 participants (via telephone, Skype or Microsoft Teams) to gain a deeper understanding on social isolation and loneliness. If you are interested in being contacted for an interview (approximately 30 minutes), please enter contact information and we will be in touch; **if you are not interested please click next to register your responses**. We are expecting a large volume of responses, therefore we may not be able to follow up with you. All information will be confidential.

- Name (1) __________________________________________________
- Email Address (2) __________________________________________________
- Number (3) __________________________________________________

End of Block: Default Question Block
